# Natural Compounds as Regulators of NLRP3 Inflammasome-Mediated IL-1*β* Production

**DOI:** 10.1155/2016/5460302

**Published:** 2016-09-08

**Authors:** József Tőzsér, Szilvia Benkő

**Affiliations:** ^1^Department of Biochemistry and Molecular Biology, Faculty of Medicine, University of Debrecen, Debrecen, Hungary; ^2^Department of Physiology, Faculty of Medicine, University of Debrecen, Debrecen, Hungary

## Abstract

IL-1*β* is one of the main proinflammatory cytokines that regulates a broad range of immune responses and also participates in several physiological processes. The canonical production of IL-1*β* requires multiprotein complexes called inflammasomes. One of the most intensively studied inflammasome complexes is the NLRP3 inflammasome. Its activation requires two signals: one signal “primes” the cells and induces the expression of NLRP3 and pro-IL-1*β*, while the other signal leads to the assembly and activation of the complex. Several stimuli were reported to function as the second signal including reactive oxygen species, lysosomal rupture, or cytosolic ion perturbation. Despite very intensive studies, the precise function and regulation of the NLRP3 inflammasome are still not clear. However, many chronic inflammatory diseases are related to the overproduction of IL-1*β* that is mediated* via* the NLRP3 inflammasome. In this review, we aimed to provide an overview of studies that demonstrated the effect of plant-derived natural compounds on NLRP3 inflammasome-mediated IL-1*β* production. Although many of these studies lack the mechanistic explanation of their action, these compounds may be considered as complementary supplements in the treatment of chronic inflammatory diseases, consumed as preventive agents, and may also be considered as molecular tools to study NLRP3 function.

## 1. Introduction

Inflammation is an important host response triggered by invading pathogens or damaged tissues, a response that is aimed at diluting or destroying the pathogens or isolating the involved site. Moderate inflammatory response contributes to the host defense by removing pathogens or aiding in the repair of damaged tissue. However, uncontrolled or prolonged inflammation may promote further tissue damage and could lead to serious disorders due to the overproduction of inflammatory cytokines.

Among inflammatory mediators, IL-1*β* is a master regulatory cytokine, functioning at several levels of immune responses, such as activation of cells to produce other inflammatory cytokines and chemokines, induction of endothelial cells to express cell membrane adhesion molecules, or assisting in the polarization of human Th17 cells [1–3]. Furthermore, it also participates in a variety of physiological processes, such as the regulation of synaptic plasticity and memory processes, in addition to participating in pain development [4–7].

The production of IL-1*β* requires a multiprotein complex called inflammasome. One of the most intensively studied inflammasomes is the NLRP3 inflammasome that contains NLRP3 sensor, ASC adaptor, and caspase-1 protease [8]. The presence of NLRP3 inflammasome has been shown not only in immunocompetent cells but also in cells responsible for various physiological functions, such as muscle cells, neurons, or endocrine cells. Upon stimulation, NLRP3 inflammasome components assemble into large cytoplasmic complexes, and the activation of caspase-1 eventually leads to the maturation and secretion of IL-1*β*. Besides cytokine production, NLRP3 inflammasome activation may also be accompanied by caspase-1-mediated rapid cell death, which is known as pyroptosis [9].

## 2. Mechanism of NLRP3 Inflammasome Activation

**Figure 1:**
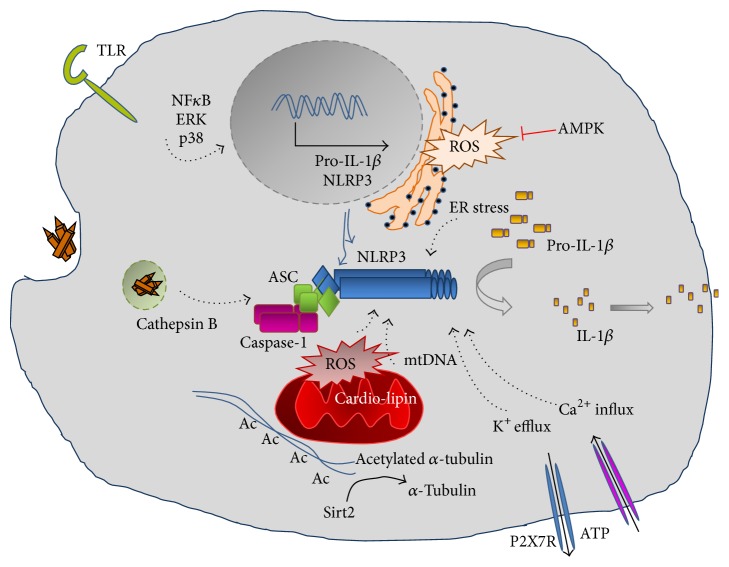
Basic mechanisms of NLRP3 inflammasome activation.

NLRP3 inflammasome can be activated by a broad range of stimuli that belong either to pathogen-associated molecular patterns (PAMPs) released during viral, bacterial, fungal, or protozoa infection [10–13] or to danger-associated molecular patterns (DAMPs) of endogenous or exogenous origin, like extracellular ATP, reactive oxygen species (ROS), cholesterol, monosodium urate (MSU) crystals, amyloid beta (A*β*) plaques, silica, or asbestos [14–20] (Figure 1).

Due to the multiple functions of IL-1*β*, production of this cytokine is tightly regulated, requiring two signals. The first signal, called “priming,” is mediated through PAMPs recognized by specific receptors like TLRs, activating signal transduction pathways that induce the expression of the inflammasome components as well as that of the inactive cytokine precursor pro-IL-1*β*. Signaling pathways through NF*κ*B, p38, and ERK1 have all been associated with the expression of NLRP3 and pro-IL-1*β* [21–23]. The second signal, provided by DAMPs or PAMPs, leads to the assembly of the inflammasome complex that is followed by activation of caspase-1 and the cleavage and secretion of active IL-1*β* [24, 25]. Considering the diversity of the second signals sensed by NLRP3, it is highly unlikely that NLRP3 is capable of interacting directly with chemically different activators. It is more likely that NLRP3 senses a general signal that induces the sequential events of inflammasome activation. Several cellular mechanisms were reported as requirements for activation, including intracellular release of oxidized mitochondrial DNA (mtDNA), increased intracellular Ca^2+^ concentration, decreased intracellular cAMP level, or pore formation by bacterial toxins [26–28]. Common cellular events are assumed to be critical for the inflammasome activation, including ROS formation, potassium (K^+^) efflux, and cathepsin B leakage from lysosomes [29]. However, due to the controversial results published in the field, the precise mechanism that mediates NLRP3 inflammasome activation is still not known. It is conceivable that more than one single requirement needs to be fulfilled, and these requirements may depend on the activating stimuli, also the* per se* characteristics of the stimulated cell.

### 2.1. Reactive Oxygen Species

Several NLRP3 inflammasome activating stimuli like nigericin or ATP induce oxidative stress and the production of intracellular ROS [15]. The source of ROS is still unclear, as some of the studies reported the role of NADPH oxidase in ROS generation [30], while other studies proved that mitochondrial dysfunction or ER stress leads to ROS production. As a consequence of mitochondrial dysfunction, oxidized mtDNA is released which can activate NLRP3 inflammasome [26], eventually leading to IL-1*β* production. It was also shown that, in case of increased cytoplasmic ROS, reduced thioredoxin (TRX) becomes oxidized and dissociates from thioredoxin binding protein (TXNIP) that in turn binds to and activates NLRP3. It was also shown that the expression of TXNIP is upregulated during ER stress, which also induces NLRP3 activation [31]. During oxidative stress, misfolded proteins accumulate, which are sensed by the ER membrane-bound inositol-requiring enzyme 1 (IRE1*α*) and protein kinase RNA-like endoplasmic reticulum kinase (PERK). In turn, they initiate ER stress response, activate NF*κ*B pathways, and induce the upregulation of TXNIP expression [32]. The role of nuclear factor- (erythroid-derived 2) like 2 (Nrf2) transcription factor-mediated antioxidant signaling pathway has also been reported as a negative regulator of NLRP3 inflammasome, as disruption of Nrf2 leads to increased production of IL-1*β* [33]. Recent works highlight the importance of metabolic changes such as in glycolysis and oxidative phosphorylation that accompany cell activation and influence ROS production [34, 35]. Moreover, ROS influences signal transduction pathways such as NF*κ*B; thus, besides NLRP3 inflammasome activation, it is also thought to play a role in the “priming” step [36].

### 2.2. Ion Flux and Perturbation

Perturbation of cytosolic ion concentration, such as K^+^ and Ca^2+^, is a common result of several NLRP inflammasome activating stimuli, as many activators were shown to directly induce ion fluxes. Nigericin or the activation of ATP-sensitive P2X7 receptor induces rapid efflux of K^+^ [37–39], and K^+^ efflux has been proven to act as a common signal to trigger NLRP3 inflammasome activation [40]. Furthermore, several pore forming toxins result in an increased intracellular Ca^2+^ that triggers NLRP3 activation [41]. Nevertheless, changes in intracellular ion concentration would also stimulate other inflammasome complexes, which argues against ion flux as the exclusive cause for NLRP3 inflammasome activation.

### 2.3. Lysosomal Rupture and Cathepsin B Release

Destabilization of the acidic lysosomal compartment and release of lysosomal protease cathepsin B into the cytoplasm during phagocytosis of large particles or crystals, such as silica, asbestos, uric acid, or beta-amyloids, have also been implicated in the activation of the NLRP3 inflammasome [42]. Furthermore, phagocytosis could also induce K^+^ efflux. Additionally, lysosomes contain high concentration of Ca^2+^, and lysosomal rupture results in the release of Ca^2+^ into the cytosol, triggering further Ca^2+^ release from the ER. However, the molecular details and connections of these events are not yet clarified. In conclusion, although NLRP3 inflammasome is the most intensively studied inflammasome complex, it is still unclear which mechanisms are responsible for its activation.

### 2.4. Mitochondrial Dysfunction

In response to infection or endogenous stimuli, the perturbation of intracellular ROS, K^+^, or lysosomal stability leads to mitochondrial dysfunction and to the release of mitochondrial DAMPs such as mtDNA, cardiolipin, or dynamin-related protein 1 (Drp1) [43]. It was shown that due to increased mtROS level, released mtDNA is oxidized and activates NLRP3 inflammasome [26]. Cardiolipin is a non-bilayer-forming phospholipid found in bacteria and in the inner mitochondrial membrane of eukaryotes, and its translocation from the inner membrane to the outer membrane was shown to activate NLRP3 inflammasome downstream of mtROS [44]. It was also reported that GTPase Drp1, that is needed for the fragmentation and aggregation of mitochondria, also induces NLRP3 inflammasome activation and IL-1*β* secretion in response to VSV infection but not to ATP or nigericin [45]. Moreover, mitofusin, an outer mitochondrial GTPase that regulates mitochondrial fusion, was also described as an NLRP3 activator during viral infection [46]. Recently, it was described that the mitochondrial antiviral signaling protein (MAVS) located in the mitochondrial membrane mediating interferon responses also induces NLRP3 inflammasome activation [47]. These reports demonstrate that the mitochondrion is a complex regulator of cytosolic homeostasis and a central platform for NLRP3 inflammasome activation.

### 2.5. Autophagy

Autophagy is an evolutionarily conserved mechanism to maintain cellular homeostasis by selectively eliminating damaged or aging organelles, microbes, and ubiquitinated proteins, through the formation of autophagosomes and using lysosomal degradation. Lines of evidence show that autophagy suppresses organelle stress-induced activation of NLRP3 inflammasome. Furthermore, it was reported that inducers of autophagy also induce the autophagosomal engulfment of IL-1*β* and its lysosomal degradation [48]. It was also shown that damaged mitochondria accumulate in autophagy-deficient macrophages that in turn lead to increased intracellular ROS and to the release of mtDNA [49].

### 2.6. Spatial Location of NLRP3

Originally, it was shown that activated NLRP3 inflammasome localizes to the mitochondria. However, recently, it was described that during inflammasome activation ER-associated NLRP3 colocalizes with the mitochondria-associated ASC to the perinuclear space* via* a microtubule-mediated organelle transport. It was shown that, upon cell activation, the decreased intracellular NAD^+^ level leads to the inhibition of sirtuin 2 (SIRT2) deacetylase, resulting in the accumulation of acetylated alfa-tubulin triggering a dynein-dependent transport of mitochondria to the perinuclear region, into the close proximity to the ER [50].

## 3. NLRP3 Inflammasome and Related Diseases

Understanding the steps and mechanism of NLRP3 inflammasome activation would be of crucial importance for the treatment of several diseases in which the inflammasome complex has been implicated. By producing IL-1*β* inflammatory cytokine and inducing pyroptosis, the primary function of NLRP3 inflammasome is to protect the host from invading microorganisms [78]. However, nonmicrobial compounds of either endogenous (self-derived) or exogenous (environmental) origin are also effective inducers of NLRP3 inflammasome activation and lead to sterile inflammation, allergic responses, or other forms of inflammation [79]. For example, studies have shown that NLRP3 may be implicated in Alzheimer's disease, suggesting that misfolded A*β* proteins form aggregates that lead to the activation of NLRP inflammasome [80, 81]. In gout, MSU crystals from purine degradation form deposits in different tissues and specifically activate NLRP3 inflammasome [14]. Environmental particulates such as inhaled asbestos and silica also activate NLRP3 inflammasome, and the high concentration of IL-1*β* is involved in the development of asbestosis and silicosis, two progressive pulmonary diseases leading to fibrosis [16, 82]. NLRP3 inflammasome has also been related to allergic responses to a variety of allergens, such as aluminum, dust mite, or ragweed pollen; however, the molecular details of these responses are still not clear [83–85].

Other studies associated NLRP3 inflammasome function with metabolic syndrome and type 2 diabetes, as NLRP3 deficient mice were protected from high fat diet- (HFT-) induced inflammation, glucose intolerance, insulin resistance, and obesity [86, 87].

The dysregulated production of IL-1*β* by the NLRP3 inflammasome is the main reason for the development of Cryopyrin-Associated Periodic Syndromes (CAPS) which is caused by a mutation in NLRP3 gene. CAPS is an autoinflammatory disorder rather than an autoimmune one, as symptoms are mediated by the innate immune system, mainly by monocytes and macrophages that produce huge amount of IL-1*β*. Behcet's disease (BD) is another chronic autoinflammatory disorder of unknown etiology, and increased IL-1*β* production has been noted as a central player in the pathogenesis of this disease. Studies demonstrated increased ROS production and, consequently, increased NLRP3 function in these patients, suggesting an important role for NLRP3 inflammasome in mediating cytokine production [88]. In addition to autoinflammatory diseases, NLRP3 inflammasome function has also been implicated in the development of autoimmune diseases like rheumatoid arthritis (RA) [89]. RA is a common inflammatory disease affecting small joints, and our knowledge about its pathogenesis is still incomplete. Nevertheless, elevated levels of IL-1*β* and high expression of NLRP3 were detected from the serum and synovial tissue as well as in macrophages of RA patients [90].

At present, the role of NLRP3 inflammasome and IL-1*β* in cancer is highly controversial. The microenvironment of tumors is characterized by an inflammatory milieu that helps tumor survival with growth hormones, endothelial activation, and angiogenesis that leads to metastasis accompanied by immune suppression. IL-1*β* participates in each of these mechanisms by stimulating the expression of TNF*α*, MMPs, VEGF, ICAM-1, VCAM, and so forth. Cancer cells, like melanoma or myeloma, as well as tumor-associated macrophages and dendritic cells, were shown to contribute to the IL-1*β* production that helps survival and growth of tumor cells; furthermore, excessive production of IL-1*β* can recruit immunosuppressive cells like myeloid-derived suppressors cells (MDSCs) [91]. On the other hand, it was also shown that NLRP3-induced IL-1*β* production boosts T cell function in patients receiving chemotherapy [92].

**Figure 2:**
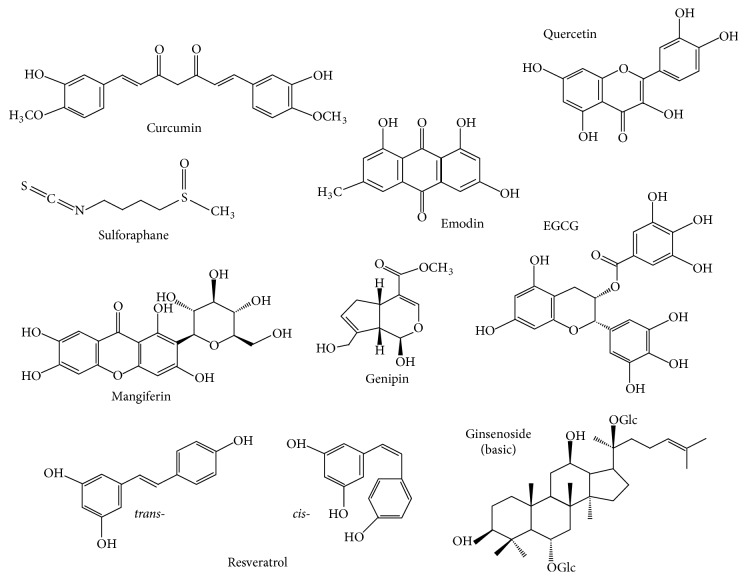
Chemical structure of natural compounds influencing NLRP3 inflammasome activation.

The classical medical approach to treating the above-mentioned diseases involves the use of synthetic drugs developed against individual elements of the signaling pathway, the IL-1*β* cytokine, or the IL-1*β* receptor, as reviewed in Ozaki et al., 2015 [93]. However, today, in the health-cautious life era, the use of botanical and natural compounds has gained popularity. While a few decades ago the use of natural compounds was based exclusively on empirical experiences, today, with the highly developed molecular biological, high throughput methods, many molecular elements of their action have already been identified. However, fundamental questions remain to be answered, including bioavailability, effective doses, body concentrations, cross-reactivity, half-life and degradation, and synergistic effect of compounds as well as carefully designed clinical trials. The aim of the present review is to provide an overview of these plant-derived natural compounds that would support the use of medicinal plants (Figure 2). As a variety of compounds can influence inflammasome activation and function, we have focused on natural compounds that have been in long term use in traditional medicine; therefore, their safety and overall effect are mostly established. However, these compounds typically influence a multitude of pathways, and the exact molecular mechanisms of their beneficial actions are not completely clarified (Table 1).

## 4. Natural Compounds Affecting NLRP3 Inflammasome Activation

### 4.1. *Aloe vera*

*Aloe vera* is a medical plant used traditionally in diverse therapeutic applications. The gel of* Aloe vera* has been reported to stimulate wound-healing and skin hydration, induce hematopoiesis, and possess antidiabetic, anticarcinogenic, antimicrobial, and antioxidant as well as anti-inflammatory activities. Over 75 active components have already been identified in* Aloe vera* leaf gels [94], and some of them have been implicated as immunomodulatory compounds based on animal studies. In a mouse sepsis model and in a human colorectal mucosa model, treatment with* Aloe vera* significantly inhibited the elevation of TNF*α*, IL-6, and IL-1*β* levels [95, 96]. Studying human THP-1 cells and human monocyte-derived macrophages, it was found that* Aloe vera* treatment significantly reduced LPS-induced IL-1*β* production [51].* Aloe vera* inhibited the expression of pro-IL-1*β*, NLRP3, and caspase-1 as well as that of the P2X7 receptor in the LPS-induced primary macrophages. Furthermore, LPS-induced activation of signaling pathways, such as NF-*κ*B, p38, JNK, and ERK, were inhibited by* Aloe vera* in these cells [51].

Aloe emodin is an anthraquinone, present naturally in* Aloe* leaves. It has been shown to promote natural killer cell activity and macrophage phagocytosis in tumor [97]. Emodin itself appears to have some protective effect in the inflammatory response. Recently, it was shown that emodin attenuated nigericin-, ATP-, and silica-induced IL-1*β* secretion from LPS-activated murine bone marrow-derived macrophages (BMDMs). It was also shown that mice treated intraperitoneally with emodin showed higher survival rates than control mice injected with LPS alone, indicating that emodin can ameliorate the severity of NLRP3 inflammasome-mediated disease symptoms* in vivo* [52]. However, further molecular details of the inhibitory effect are yet to be discovered.

### 4.2. Curcumin

Curcumin is a lipid soluble polyphenol, a yellow pigment isolated from the rhizomes of* Curcuma longa* (turmeric), but also found in other plants like ginger. It is widely used in food coloring and flavoring and it is also added to cosmetics. It has gained attention in recent years for its multiple pharmacological properties, being antioxidant, anti-inflammatory, and antimicrobial, as well as for its therapeutic potential in cancer, autoimmune, metabolic, pulmonary, cardiovascular, and neurological diseases (reviewed in [98]).

Brain ischemia is known to induce ER stress and inflammatory responses leading to neuronal damage [99, 100]. In a recent study, glucose deprivation or hypoxia was reported to strongly induce the production of glutamate and IL-1*β* in mouse hippocampus [53]. It was shown that pretreatment of the mouse hippocampus with curcumin reduced IL-1*β* production, and this effect was attenuated by 5′-AMP-activated protein kinase (AMPK) inhibitor, suggesting the possible involvement of AMPK. The authors also found that curcumin attenuated glutamate-induced phosphorylation of PERK and IRE1*α*; the transmembrane sensors of ER stress that mediate inflammatory signals. Moreover, using a neuroblastoma cell line, they showed that curcumin inhibited glutamate-induced ROS generation, as well as reduced glutamate-induced TXNIP expression. As a possible molecular mechanism, they found that, in mice hippocampus, glutamate stimulation increased NLRP3 expression and the cleaved form of caspase-1 enzyme, while curcumin attenuated NLRP3 and cleaved caspase-1 expression. The production of IL-6, a downstream target of IL-1*β*, was also inhibited by curcumin treatment. In addition, curcumin effectively attenuated mitochondrial function and prevented caspase-3 activation in hippocampus exposed to glutamate stimulation, effectively preventing glutamate-induced cell apoptosis. Furthermore, in rats, curcumin administration attenuated ischemic brain injury resulting from middle cerebral artery occlusion (MCAO). Based on these findings, curcumin activates AMPK that inhibits glutamate-induced ER stress and ROS production, thus inhibiting TXNIP-induced NLRP3 inflammasome activation and ultimately reducing IL-1*β* production in mouse hippocampus limiting brain injury.

Macrophages are popular models for the study of NLRP3 inflammasome activation. In another study performed on mouse macrophage cell line J774A.1 and peritoneal macrophages, curcumin was shown to strongly inhibit IL-1*β* secretion triggered by LPS plus nigericin, aluminum, ATP, or MSU [54]. Preincubation with curcumin prevented nigericin-induced intracellular potassium level decrease, attenuated lysosome damage and cathepsin B leakage, and blocked high mobility group box 1 (HMGB-1) release; needless to say, all of these are components that may induce NLRP3 inflammasome activation. In BMDMs, curcumin inhibited nigericin- or aluminum crystal-induced ROS production. The authors did not find obvious inhibitory effect of curcumin on JNK and p38 phosphorylation enhanced by nigericin treatment in the LPS-primed macrophages; however, they found that ERK1/2 phosphorylation was dramatically downregulated. In a mouse model of LPS-induced septic shock, administration of curcumin significantly decreased ROS level and cathepsin B leakage in peritoneal macrophages; additionally, peritoneal HMGB-1 and IL-1*β* concentrations were downregulated, resulting in a reduced LPS-induced splenomegaly. These data suggest that the observed reduction of secreted mature IL-1*β* by curcumin is primarily attributed to the inhibition of NLRP3 inflammasome activation.

### 4.3. EGCG

Epigallocatechin-3-gallate (EGCG) is the major bioactive polyphenol in green tea that has a well-documented antioxidant activity, as it functions as a free-radical scavenger. EGCG was reported to inhibit NF*κ*B activation and the subsequent expression of several inflammatory molecules such as inducible nitric oxide synthase (iNOS), matrix metalloproteinases (MMPs), IL-6, and TNF*α*. Besides the anti-inflammatory role, EGCG was also thought to possess antitumor effect [101].

In unilateral ureteral obstruction mice model, renal inflammation has been linked to the activity of NLRP3 inflammasome and the overproduction of IL-1*β*. Furthermore, the role of NLRP3 inflammasome was proven in a variety of human nondiabetic and chronic kidney diseases [102]. It was reported that EGCG can prevent the development of lupus nephritis when administered to NZB/W F1 mice, the classical model of systemic lupus erythematosus [55]. These studies showed that EGCG treatment activated Nrf2 antioxidant pathway, induced the expression of Nrf2 downstream enzymes like NAD(P)H dehydrogenase (quinone) 1 (NQO1) and heme oxygenase 1 (HO-1), and reduced renal oxidative stress. Furthermore, EGCG inhibited renal NLRP3 inflammasome activity, and the expressions of NLRP3, caspase-1, and IL-1*β* were also reduced, possibly due to the EGCG-induced attenuation of NF*κ*B pathway activity.

Very recently, the beneficial effect of EGCG was reported in a contrast-induced nephropathy (CIN) rat model [56]. CIN is a common iatrogenic cause of acute kidney injury (AKI) after exposure to iodinated contrast medium, characterized by oxidative stress and inflammation [103]. Similar to the previous study, they found that EGCG treatment reduced oxidative stress, and increased Nrf2 and HO-1 expression. They also found that EGCG reduced the elevated IL-1*β* secretion and NLRP3 protein expression. The molecular mechanisms of the observed results, however, were not studied.

### 4.4. Genipin

Genipin is a water-soluble bifunctional cross-linking reagent that was initially isolated from the Chinese medicinal plant* Gardenia jasminoides* (common name jasmine), and it has been used for centuries as a herbal medicine to protect the liver and gallbladder. It possesses antihypertensive, antibleeding, and antiswelling properties, in addition to exhibiting anti-inflammatory characteristics. Chemically, it is an aglycone, derived from an iridoid glycoside called geniposide that is enzymatically hydrolyzed to genipin by intestinal bacteria when administered* per os* [104]. Genipin was reported to inhibit mitochondrial uncoupling protein 2 (UCP2) as well as superoxide-activated UCP2-mediated proton leak occurring in a variety of diseases [105].

Recently, it was shown that overexpression of UCP2 caused a significant increase in NLRP3 expression in THP-1 cells, and this was significantly inhibited by the administration of genipin, while the levels of the adaptor protein ASC did not change [57]. Interestingly, genipin treatment attenuated IL-1*β* levels in LPS-primed cells treated with ATP; however, this effect was not observed in cells treated with nigericin. In addition, it was also shown that inhibition of ATP-mediated inflammasome activation by genipin does not alter the production of other proinflammatory cytokines such as IL-6 and TNF*α*.

In another recent study, in LPS-primed murine BMDMs, genipin dramatically inhibited IL-1*β* secretion of LPS-primed cells following ATP, nigericin, MSU crystals, or* Listeria monocytogenes* treatment [58]. Furthermore, genipin also inhibited the* Salmonella typhimurium*-induced secretion of IL-1*β*, which is mediated* via* the activation of NLRC4 inflammasome. Similar to the previous study, LPS-dependent TNF*α* secretion was not impaired by genipin. Importantly, genipin treatment did not inhibit the expression of pro-IL-1*β*, pro-caspase-1, or ASC proteins; however, it inhibited NLRP3-dependent ASC oligomerization in addition to IL-1*β* secretion and caspase-1 activation. In contrast to its inhibitory effect on NLRP3-mediated ASC oligomerization, genipin did not affect NLRC4-mediated formation of ASC speckles, while NLRC4-mediated caspase-1 activation was significantly diminished. These data indicate that the inhibitory effect of genipin on NLRC4 inflammasome activation is independent of ASC.

### 4.5. Ginseng

White ginseng is the naturally dried form of ginseng (*Panax ginseng*), while red ginseng is made by steaming and drying fresh ginseng root. Red ginseng has been used for centuries as an immune-modulator and has also been used as an adjuvant in the treatment of infectious and metabolic diseases. Korean red ginseng contains various active components, including saponins such as ginsenosides and nonsaponins such as polysaccharides, peptides, fatty acids, and mineral oils. Ginsenosides are the major active components of ginseng. They reduce the production of inflammatory cytokines by inhibiting NF*κ*B signaling [106]. They also have amphiphilic nature and are able to intercalate into the plasma membrane and influence membrane fluidity and function.

It was shown that red ginseng extract (RGE) inhibited ATP-, nigericin-, and aluminum-induced IL-1*β* secretion in LPS-primed BMDMs and THP-1 cells and ameliorated lethality in LPS-induced septic shock model of mice [59]. Furthermore, RGE attenuated IL-1*β* secretion in dsDNA-transfected macrophages. Similar results were obtained for ginsenosides. Furthermore, it was also shown that RGE acted as an inhibitor of both NLRP3 and AIM2 inflammasome activations. It was also demonstrated that RGE attenuated the secretion of the active form of caspase-1 (p20).

It was previously reported that endogenous NO produced by iNOS inhibits NLRP3 inflammasome activation* via* the S-nitrosylation of NLRP3 protein [107, 108]. A recent study reported that ginsenosides negatively regulates LPS-induced septic shock in mice by a mechanism involving inhibition of iNOS expression and the subsequent NO production [60]. It was shown that ginsenosides reduced NO level, iNOS expression, and ROS level in peritoneal macrophages and BMDMs after LPS stimulation. They also found that ginsenoside treatment attenuated S-nitrosylation of NLRP3 protein, which resulted in increased IL-beta secretion in LPS-activated macrophages.

### 4.6. Mangiferin

Mangiferin is a water-soluble, natural polyphenol with a C-glycosylxanthone structure. The primary source of mangiferin is the mango tree (*Mangifera indica*); however, it is also present in some medicinal herbs, in* Iris unguicularis*, and in the honeybush, a popular herbal tea from South Africa. It possesses a wide spectrum of beneficial effects on health, being used as an analgesic, antidiabetic, antisclerotic, antimicrobial, and antiviral, as well as being praised for its anti-inflammatory effects. It has been shown to regulate AMPK activity, and, in a recent work, it was reported that, in human endothelial cells exposed to high glucose, mangiferin reduced IL-1*β* secretion [61]. Furthermore, mangiferin attenuated IRE1 phosphorylation and reduced ROS production, and it decreased TXNIP expression and inhibited ER stress. Moreover, a decrease in NLRP3 expression and IL-1*β* production was also demonstrated.

Very recently, the protective role of mangiferin was described in LPS plus D-galactosamine- (D-GalN-) induced acute liver injury [62]. The authors showed that mangiferin reduced IL-1*β* secretion and ROS production, and it also inhibited iNOS expression but induced Nrf2 and HO-1 expression. Furthermore, it attenuated NLRP3, ASC, and caspase-1 (p10) expression and the subsequent IL-1*β* production.

### 4.7. Propolis

Propolis is a resinous material, produced by bees (*Apis mellifera L*.) through the mixing of secretions and enzymes from their hypopharyngeal glands with the buds of plants and wax. A few hundred compounds have been detected in raw propolis, including polyphenols, terpenoids, steroids, sugar, and amino acids. Propolis composition is largely influenced by botanical origins and geographical location, as well as by collection season. One of the most known biological activities of propolis is related to its anti-inflammatory effect. Artepillin C is a simple phenol composed of a single ring with two prenyl groups (3,5-diprenyl-4-hydroxycinnamic acid) and is the major biologically active phenolic ingredient identified in green propolis derived from Southeast Brazil [109].

The role of artepillin C was studied on the oxidative metabolism of PMA-stimulated RAW264.7 macrophages [63]. It was shown that artepillin C significantly inhibited ROS release upon the NLRP3 activator nigericin treatment. It was also suggested that artepillin C significantly contributed to the modulation of chemokine-mediated inflammation, as it significantly downregulated the release of IL-1*β*, IL-12p40, and TNF*α* by LPS + IFN*γ*-treated RAW264.7 cells, whereas the synthesis of IL-1*α* and IL-6 was not affected. Besides nigericin-mediated IL-1*β* production, propolis also inhibited* Legionella pneumophila*-induced IL-1*β* cytokine secretion in LPS-primed BMDMs. This indicates that both NLRP3 and NLRC4 inflammasome functions may be regulated by propolis; however, further studies are required to clarify the detailed molecular mechanism of the observed results.

### 4.8. Quercetin

Quercetin, a kind of flavonoid found in a variety of vegetables, beverages, fruits, and herbs, exhibits anti-inflammatory and antioxidant properties. Quercetin was reported to reduce inflammation in visceral adipose tissue, primary human adipocytes, and the kidney of fructose-fed rats. It was also shown to prevent diabetic kidney injury in rats. However, the mechanisms of the action of quercetin were not clarified.

It has been described that quercetin attenuated fructose-induced hyperuricemia and renal dysfunction. In a study, using streptozotocin- (STZ-) induced diabetic nephropathy rat model leading to hyperuricemia and dyslipidemia, quercetin was found to block NLRP3 inflammasome activation [64]. It was shown that the STZ-induced expression of inflammasome components was restrained, and IL-1*β* secretion was reduced by quercetin treatment. Similar results were obtained studying inflammatory responses in fructose-induced hyperuricemia in rat [65]. They showed that fructose significantly elevated the expression of renal NLRP3, ASC, and caspase-1 expression, while quercetin treatment ameliorated the expression of these proteins. Based on their findings, it was suggested that fructose-mediated renal NLRP3 inflammasome activation and proinflammatory cytokine overproduction may be associated with the impaired cross talk between renal JAK2-STAT3 and PPAR-*α* signaling pathway that in turn regulate renal inflammation.

Furthermore, it was shown that quercetin markedly inhibited the overexpression of hepatic TXNIP, in addition to reducing the production of ROS and the activation of NLRP3 inflammasome, consequently resulting in decreased IL-1*β* secretion [66, 110]. They also showed that the decreased caspase-1 activity downregulated the caspase-1-dependent activation of SREBPs that are involved in fatty acid and cholesterol synthesis. They suggested that regulation of the TXNIP-NLRP3-SREBPs pathway by quercetin may partially mediate the alleviation of hepatic steatosis in these diabetic rats.

It was also shown that quercetin treatment regulated AMPK pathway activation, glutamine-glutamate cycle dysfunction, and TXNIP overexpression; moreover, it suppressed NF*κ*B pathway, eventually leading to impaired NLRP3 inflammasome activation and IL-1*β* production in the hypothalamus of high fructose- (HF-) fed rats [67].

Using human umbilical vein endothelial cells, another group reported that quercetin suppressed palmitate- (PA-) induced IL-1*β* and IL-6 induction [68]. Downregulation of NLRP3 induction was probably partially mediated* via* the inhibition of ROS production and TXNIP expression; quercetin also inhibited IKK*β* activation by attenuating its phosphorylation. Furthermore, quercetin treatment protected cells against PA insult and significantly reduced the number of apoptotic cells. Moreover, quercetin ameliorated ER stress-induced endothelial dysfunction through the activation of AMPK.

NLRP3 inflammasome is also recognized to have an important role in the spinal cord tissue after traumatic spinal cord injury (SCI), and targeting the NLRP3 inflammasome can inhibit neuroinflammation, thereby improving functional recovery in SCI rats [111].

Very recently, in a rat model of SCI, quercetin administration was shown to significantly improve functional recovery [69]. Histopathological changes of the spinal cord, such as congestion, edema, neutrophil infiltration, and structural disruption, were inhibited by quercetin administration. To formulate a molecular mechanism, the authors showed that quercetin administration significantly reduced inflammatory cytokine IL-1*β*, IL-18, and TNF*α* levels; it also reduced the NLRP3, ASC, and active caspase-1 protein expression and ROS production. However, further studies are needed to better characterize the mechanisms of action underlying the beneficial effects of quercetin in inflammation and immunity.

### 4.9. Resveratrol

Resveratrol is a polyphenol present in the skin of grapes and mulberry, commonly used in red wine production. The beneficial effects of resveratrol are thought to be mediated, in part, by its antioxidant and anti-inflammatory properties, exhibiting beneficial effects in several diseases [112]. Resveratrol exists as two isomers,* cis*- and* trans*-resveratrol. The* cis* isomer is thought to be produced naturally during grape fermentation as a result of isomerization of the* trans* isomer by yeast isomerases. Furthermore,* cis*-resveratrol can be obtained by exposure of the* trans* isomer to sunlight. Several studies have shown that* trans*-resveratrol exhibits a wide range of biological activities, including anti-inflammatory, antioxidant, anticarcinogenic, antiaging, antiplatelet aggregation, immune-modulatory, and chemoprevention effects. However, only few studies have been performed to examine the biological effects of the* cis* isomer. Studies on* cis*-resveratrol have demonstrated that it also possesses anticancer, antimicrobial, and antiplatelet aggregation activities.

One of the biological roles of resveratrol is to initiate the activation of sirtuin 1 (Sirt1) is a deacetylase that modifies and inactivates inflammatory genes. At high doses, resveratrol has been shown to activate AMPK; which is involved in the negative regulation of the NLRP3-inflammasome [113]. It was also shown that ionizing radiation-induced IL-1*β* secretion by human mesenchymal stem cells (MSCs), an effect that was effectively abrogated by pretreatment with resveratrol [70]. Radiation-induced increase in IL-1*β* occurs* via* the NLRP3 inflammasome activation; however, resveratrol treatment attenuated the expression of NLRP3. It was also demonstrated that resveratrol treatment upregulated the expression of Sirt1. In conclusion, it is suggested that Sirt1 inhibits NF*κ*B transcriptional activity through deacetylation that in turn limits the transcription of NLRP3.

In another study, it was shown that* cis-*resveratrol pretreatment significantly inhibited IL-1*β* secretion in THP-1 cell culture supernatants; interestingly,* cis*-resveratrol induced IL-18 secretion under these conditions [71]. Accordingly,* cis*-resveratrol significantly reduced IL-1*β* mRNA expression; in contrast,* cis*-resveratrol used at the same concentration increased IL-18 mRNA expression. Furthermore,* cis*-resveratrol increased the mRNA and protein levels of NLRP1 and NLRP3, indicating that the inhibitory effect of* cis-*resveratrol on IL-1*β* secretion is not due to the downregulation of NLRP1, NLRP3, or ASC expression. Similar results were obtained on LPS-primed J774A.1 macrophages where resveratrol inhibited the ATP-induced IL-1*β* secretion; however, in this study, the authors did not find changes in the expression of NLRP3 [72], showing that the resveratrol did not inhibit the priming signal of NLRP3.

Huang et al. suggested that the inhibitory effect of* cis*-resveratrol on IL-1*β* secretion may be due to the reduced expression of purinergic receptor P2X7R and the ER stress marker GRP78, which leads to suppression of ROS production and reduced caspase-1 and caspase-4 activation. It was found that* cis*-resveratrol also inhibited cyclooxygenase- (COX-) 2 expression, which may be responsible for the observed decrease of PGE2 production. Furthermore, inhibition of pro-IL-1*β* and COX-2 expression may occur by downregulating the activation of the p38 MAPK pathway [71]. Importantly, in contrast to these observations, increased phosphorylation of p38 was detected in ATP-activated J774A.1 macrophages by Chang et al., while it did not significantly increase the phosphorylation levels of ERK1/2, and it reduced the phosphorylation levels of JNK1/2. The levels of mtDNA release into the cytosol were significantly reduced by resveratrol, indicating that there was less mitochondrial injury in resveratrol-treated cells [72] Furthermore, the authors also showed that resveratrol increased cellular cAMP level in J774A.1 macrophages, which acts as a negative regulator of inflammatory responses and inhibits NLRP3 inflammasome activation.

The effect of resveratrol on NLRP3 inflammasome was further proved by a study showing that four weeks of resveratrol administration inhibited the expression of NLRP3 inflammasome components, IL-1, IL-6, and TNF*α*, and in livers of HF-fed mice [73]. They also showed that the regulatory effects of resveratrol on NLRP3 inflammasome may come about through the combined effects of Sirt1 and Sirt6.

In a recent study [50], it was found that resveratrol treatment successfully suppressed the maturation of both IL-1*β* and caspase-1 in response to Pam3CSK4; a TLR1/2 agonist, plus various inducers (like nigericin, ATP, MSU, and silica) of the NLRP3-inflammasome. In mouse BMDMs, however, it did not affect the production of IL-1*β* mediated by the NLRC4-inflammasome (flagellin) or the AIM2-inflammasome (dsDNA). On a mouse model of acute gout, they indicated that resveratrol reduced the amount of mature IL-1*β* and the subsequent recruitment of neutrophils into the peritoneal cavities of mice after challenge with MSU crystals. These findings indicate that resveratrol suppresses the activation of the NLRP3-inflammasome both* in vitro* and* in vivo*. As a possible mechanism, they showed that resveratrol at a substantially higher dosage successfully suppressed mitochondrial ROS production. Furthermore, resveratrol treatment prevented the accumulation of acetylated *α*-tubulin; thus,inhibiting the proximate localization of ASC and NLRP3 in macrophages.

In a very recent publication [74], the authors showed that resveratrol prevented ATP-induced NLRP3 activation and IL-1*β* production in the BV2 cell line and in septic mice as well. They also found that resveratrol treatment reduced NLRP3 and IL-1*β* expression in the hippocampus of the mice, and they suggested that the protecting effect of resveratrol was mediated* via* the increased expression of Sirt1 in BV2 cells.

Another recent publication found that glucose-based peritoneal dialysis increased mitochondrial ROS production and subsequent IL-1*β* secretion through NLRP3 inflammasome in human peritoneal mesothelial cells (HPMC). As a mechanism for its action, they showed that resveratrol induced mitophagy/autophagy of the cells* via* AMPK activation and protected cells from ROS-induced NLRP3-mediated inflammatory injury [75].

### 4.10. Sulforaphane

Sulforaphane (SFN) is a natural compound present in cruciferous vegetables such as broccoli, cabbage, Brussel sprouts, mustard, and radish. Chemically, SFN is an isothiocyanate that is known to react with sulfhydryl groups and to modify proteins at cysteine residues. Sulforaphane is produced from the precursor glucoraphanin by the enzymatic activity of myrosinase, when the cell walls of the plant are damaged, for example, during chewing. SFN has been known as a cytoprotective agent for cancer prevention or treatment, and it also has an important role in cardiovascular, neurologic, and other diseases (reviewed in [114]). SFN is a known inducer of the Nrf2 antioxidant pathway, as it activates the Nrf2 transcription factor by reacting with cysteine residues of its repressor, Keap1 [115]. In addition, it also inhibits NF*κ*B signaling [116].

It was shown that SFN inhibited IL-1*β* processing by NLRP1-, NLRP3-, NLRC4-, and AIM2- inflammasome complexes in mouse BMDMs [28]. It was also demonstrated that SFN inhibits the inflammasome in an Nrf2-independent manner, as SFN-mediated inhibition of NLRP3- and NLRC4-dependent IL-1*β* processing and secretion was not reversed in Nrf2-knockout BMDMs. When SFN was added directly to the cell lysates, it did not inhibit IL-1*β* processing, demonstrating that SFN does not directly inhibit the protease activity of caspase-1. The authors found that SFN-mediated inhibition of the inflammasomes is not affected by ROS modulation, and neither proteasome-dependent proteolysis nor* de novo* protein synthesis is required for SFN-mediated inhibition of the inflammasomes. In a peritonitis model of acute gout, SFN treatment significantly reduced MSU crystal-induced IL-1*β* production, demonstrating that SFN inhibits the NLRP3 inflammasome function* in vivo*. Nonetheless, the authors concluded that SFN inhibits multiple inflammasomes* in vitro* and* in vivo* by a mechanism independent of inflammasome priming and Nrf2 signaling; however, the exact molecular mechanism was not explored.

As SFN is able to penetrate the blood-brain barrier [117], it gained a considerable attention as a candidate for Alzheimer's disease (AD) therapy. SFN was shown to protect the brain from A*β*-induced oxidative cell death* via* activation of Nrf2 signaling cascade [118]. As A*β* fibrils have been shown to activate NLRP3 inflammasome, the question arose whether SFN may regulate this mechanism. In a very recent study, it was shown that SFN significantly downregulated IL-1*β* production and NLRP3 expression in A*β*1–42 peptide-stimulated human THP-1 cells macrophages [76]. It was found that A*β*1–42 peptide-induced STAT-1 phosphorylation was significantly attenuated by SFN. The authors showed that SFN induced Nrf2 nuclear translocation, which was subsequently accompanied by an increase in HO-1 protein levels. Furthermore, A*β*1–42 peptide-induced IL-1*β* production was diminished by siRNA-mediated knockdown of Nrf2 or HO-1. They also found that the increased proinflammatory miRNA-146a level following A*β*1–42 treatment was significantly attenuated by SFN, whereas miRNA-125b and miRNA-155 levels were not changed. These are specific proinflammatory miRNAs that were reported to be increased in both the extracellular fluid and cerebrospinal fluid of AD patients. In conclusion, SFN can suppress A*β*1–42-induced caspase-1-dependent inflammasome activation, possibly through inhibiting STAT-1 phosphorylation and activation of the Nrf2/HO-1 signaling pathway.

Also very recently, another research group reported that oral administrations of SFN suppressed the saturated fatty acid-induced NLRP3 inflammasome activation in the liver of mice [77]. They found that SFN decreased the expression of ASC, caspase-1, and IL-1*β* levels; additionally, it inhibited the activity of caspase-1 enzyme in primary hepatocytes. It was also shown that SFN reduced the palmitate-induced ROS production by the mitochondria, implying that SFN prevented mitochondrial dysfunction, and this protecting mechanism was in part mediated by the activation of AMPK. They concluded that activation of AMPK and autophagy by SFN could protect from mitochondrial dysfunction induced by saturated fatty acids, resulting in a downregulation of fat-induced activation of the NLRP3 inflammasome.

## 5. NLRP3 Inflammasome and the Xenohormesis Hypothesis

**Figure 3:**
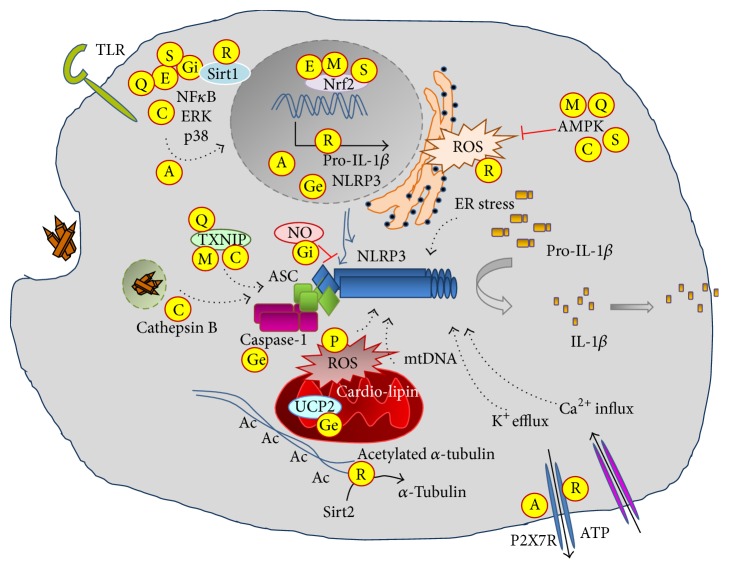
Molecular targets of natural compounds affecting NLRP3 inflammasome-mediated IL-1 production.

As it was described in detail above, polyphenols like resveratrol, curcumin, EGCG, and quercetin are potent inhibitors of NLRP3 inflammasome-mediated IL-1*β* production, typically acting at more than one element of the involved pathways (Figure 3). However, it should be noted that these polyphenols have an even much broader biological effect, as they influence a variety of pathways [119], though not all are directly connected to the function of the NLRP3 inflammasome. Importantly, more than 8000 natural polyphenols have been described [120], providing a large library of compounds as potential inflammasome inhibitors. Many of these polyphenols (such as resveratrol and curcumin) are overproduced by stressed plants, triggering pathways mimicking the effect of caloric restriction. It has been proposed that organisms, from yeast to humans, consuming stressed plants could interpret the stress signal carried by such polyphenols as a potential risk of future limitation of food availability, and, in turn, adapting for the pursuit of longevity, a theory termed as Xenohormesis [121–123]. Interestingly, the structurally unrelated resveratrol and curcumin can achieve this caloric restriction mimicking effect by activating AMPK and Sirt1 [124]. However, polyphenols are also known to have an antioxidant effect, activate glutathione S-transferases, and inhibit COX enzymes among many other effects [125, 126]. Interestingly, one of the most frequently used COX inhibitor is aspirin, a derivative of another stress-induced phytochemical, salicylic acid.

A major concern of polyphenol use in therapy is the bioavailability of these compounds. Nevertheless, typical dietary polyphenol intake is about 14 mg/kg/day, which is within the range of providing biologically active doses [127].

## 6. Concluding Remarks

Most of the time, inflammasome-mediated IL-1*β* production or caspase-mediated pyroptosis is beneficial for the host, as it protects from infection or prevents further damage. However, prolonged cytokine production, as in the case of sterile inflammation caused by endogenous danger signals, may lead to the development of autoinflammatory or metabolic diseases. Chronic inflammation compromises the host's life quality and requires sustained pharmacological and surgical treatments. The paradigm of drug discovery in Western medicine is to develop highly selective compounds against individual druggable targets. Currently, the main treatment methods for these patients are targeting IL-1*β*. However, prolonged usage of drugs may induce different side effects; furthermore, some form of medications are very expensive, which limits their widespread use.

Besides the high expenses, another drawback of traditional drug design is that many of the promising compounds fail in the early or later stages of drug development. Traditional medicine uses many plant products to treat diseases; however, the exact mechanisms behind their use is still mostly lacking, partially as a consequence of the pleiotropic effect of the active compounds, as detailed in this review. Nevertheless, clarification of the molecular mechanism of action of such natural compounds, already used in traditional medicine for quite some time, will undoubtedly aid in the production of safe and cheap candidate medications to be used in the treatment of diseases, including those appearing as a consequence of NLRP3 inflammasome-mediated IL-1*β* overproduction.

## Figures and Tables

**Table 1:** Natural compounds effecting NLRP3 inflammasome-mediated IL-1 production.

Compound	Effect	Mechanism	Target cell	Reference
*Aloe vera extract *	Reduced IL-1*β*	Reduced expression of NLRP3, procaspase-1, and P2X7R Inhibition of ERK, p38, NF*κ*B signaling	THP-1 and human MF	[51]

*Aloe emodin *	Reduced IL-1*β*	Reduced expression of NLRP3, procaspase-1, and ASC expression	BMDM	[52]

*Aloe emodin *	Enhanced survival		NLRP3-KO mice model of septic shock	[52]

*Curcumin *	Reduced IL-1*β* and caspase-1 inhibition	Reduced ER stress through AMPK	Mouse hippocampus	[53]

*Curcumin*		Reduced ROS and TXNIP expression	Neuroblastoma cells	[53]

*Curcumin *	Reduced IL-1*β* and caspase-1 inhibition	Lysosome protection	J774A.1	[54]

*Curcumin*	Reduced IL-1*β*	Reduced ROS	BMDM	[54]

*Curcumin *	Reduced IL-1*β*	Reduced ROS and lysosome damage Inhibition of ERK1/2 signaling	Mouse peritoneal MF of septic shock model	[54]

*EGCG *	Reduced IL-1*β* and caspase-1 inhibition	Reduced expression of NLRP3 and procaspase-1 and Nrf2 induction	Kidney tissue from SLE mouse model	[55]

*EGCG *	Reduced IL-1*β*	Reduced expression of NLRP3 Nrf2 induction	Rat model of contrast-induced nephropathy	[56]

*Genipin*	Reduced IL-1*β*	Reduced expression of NLRP3	THP-1	[57]

*Genipin *	Reduced IL-1*β* and caspase-1 inhibition	Reduced ASC oligomerization Induced ROS and reduced autophagy	BMDM NLRP3-KO mice	[58]

*Ginseng *	Reduced IL-1*β* and caspase-1 inhibition		BMDM, THP-1	[59]

*Ginseng *	Reduced IL-1*β*	Inhibition of iNOS and reduced NO and ROS Reduced S-nitrosylation of NLRP3 and caspase-1	RAW264.7, BMDM	[60]

*Mangiferin *	Reduced IL-1*β*	Reduced expression of NLRP3 Induced AMPK phosphorylation Reduced ROS and TXNIP expression	Endothelial cells	[61]

*Mangiferin *	Reduced IL-1*β*	Reduced expression of NLRP3, ASC, caspase-1, and iNOS Nrf2 induction	Murine primary hepatocytes	[62]

*Propolis*	Reduced IL-1*β*	Reduced ROS	BMDM	[63]

*Quercetin *	Reduced IL-1*β*	Reduced expression of NLRP3, ASC, and caspase-1	Rat model of streptozotocin- induced diabetic nephropathy	[64]

*Quercetin *	Reduced IL-1*β*	Reduced expression of NLRP3, ASC, and caspase-1	Rat model of fructose-induced hyperuricemia	[65]

*Quercetin *	Reduced IL-1*β* and caspase-1 inhibition	Reduced expression of NLRP3, ASC, and caspase-1 Reduced ROS and TXNIP expression	Hepatocytes from rat model of streptozotocin-induced diabetes	[66]

*Quercetin *	Reduced IL-1*β*	Reduced expression of NLRP3, ASC, and caspase-1 Reduced TXNIP expression and NF*κ*B signaling	Hypothalamus of fructose-fed rat	[67]

*Quercetin *	Reduced IL-1*β*	Reduced NLRP3 expression and induced AMPK Reduced ROS and TXNIP expression Inhibition of IKK*β*	Endothelial cells	[68]

*Quercetin *	Reduced IL-1*β*	Reduced expression of NLRP3, ASC, and caspase-1 Reduced ROS	Rat model of spinal cord injury	[69]

*Resveratrol*	Reduced IL-1*β*	Reduced NLRP3 expression and induced Sirt1 expression	Human mesenchymal stem cells	[70]

*Resveratrol *	Reduced IL-1*β*	Reduced expression of NLRP3 and ASC Reduced P2X7R and COX-2 expression Reduced ROS and p38 signaling	THP1	[71]

*Resveratrol *	Reduced IL-1*β*	Reduced mtDNA release and increased cAMP Increased p38 and JNK signaling	J774A.1	[72]

*Resveratrol*	Reduced IL-1*β*	Reduced NLRP3 expression and induced Sirt1 expression	Liver of high fat diet-mice	[73]

*Resveratrol *	Reduced IL-1*β* and caspase-1 inhibition	Reduced mtROS Reduced accumulation of acetylated tubulin	BMDM Mouse model of acute gout	[50]

*Resveratrol *	Reduced IL-1*β*	Reduced NLRP3 expression and induced Sirt1 expression	BV2 cells Hippocampus of septic mice	[74]

*Resveratrol*	Reduced IL-1*β*	Induction of autophagy and AMPK and reduced ROS	Human peritoneal mesothelial cells	[75]

*Sulforaphane *	Reduced IL-1*β* and caspase-1 inhibition		BMDM Peritonitis mouse model of acute gout	[28]

*Sulforaphane*	Reduced IL-1*β*	Reduced STAT1 signaling and induced Nrf2 translocation	THP-1	[76]

*Sulforaphane *	Reduced IL-1*β* and caspase-1 inhibition	Reduced expression of NLRP3, ASC, and caspase-1 Reduced ROS and reduced mitochondrial dysfunction Activation of AMPK and autophagy	Murine hepatocytes	[77]
